# MicroRNA-146a inhibition promotes total neurite outgrowth and suppresses cell apoptosis, inflammation, and STAT1/MYC pathway in PC12 and cortical neuron cellular Alzheimer's disease models

**DOI:** 10.1590/1414-431X20209665

**Published:** 2021-03-15

**Authors:** Yinghui Ma, Jiye Ye, Li Zhao, Dongmei Pan

**Affiliations:** 1 Department of Neurosurgery, Huangshi Central Hospital, Affiliated Hospital of Hubei Polytechnic University, Huangshi, China; 2 Department of Gerontology, Huangshi Central Hospital, Affiliated Hospital of Hubei Polytechnic University, Huangshi, China

**Keywords:** Alzheimer's disease, MicroRNA 146a, STAT1/MYC, Total neurite outgrowth, Cell apoptosis, Inflammation

## Abstract

This study aimed to explore the effect of microRNA (miR)-146a inhibition on regulating cell apoptosis, total neurite outgrowth, inflammation, and STAT1/MYC pathway in Alzheimer's disease (AD). PC12 and cortical neuron cellular AD models were constructed by Aβ1-42 insult. For the former model, nerve growth factor (NGF) stimulation was previously conducted. miR-146a inhibitor and negative-control (NC) inhibitor were transfected into the two cellular AD models, and then cells were named miR-inhibitor group and NC-inhibitor group, respectively. After transfection, cell apoptosis, total neurite outgrowth, supernatant inflammation cytokines, and STAT1/MYC pathway were detected. miR-146a expression was similar between PC12 cellular AD model and control cells (NGF-stimulated PC12 cells), while miR-146a expression was increased in cortical neuron cellular AD model compared with control cells (rat embryo primary cortical neurons). In both PC12 and cortical neuron cellular AD models, miR-146a expression was reduced in miR-inhibitor group compared with NC-inhibitor group after transfection. Furthermore, cell apoptosis was attenuated, while total neurite outgrowth was elevated in miR-inhibitor group compared with NC-inhibitor group. As for supernatant inflammatory cytokines, tumor necrosis factor-α, interleukin (IL)-1β, IL-6, and IL-17 levels were lower in miR-inhibitor group than in NC-inhibitor group. Additionally, STAT1 and c-Myc mRNA and protein expressions were attenuated in miR-inhibitor group compared with NC-inhibitor group. In conclusion, miR-146a potentially represented a viable therapeutic target for AD.

## Introduction

Alzheimer's disease (AD), the most prevalent neurodegenerative disorder of the elderly population, is a major global public health burden with over 26 million affected (1,2). AD is neuropathologically marked by deposition of abnormally folded amyloid-β (Aβ), tau proteins in extracellular amyloid plaques, and intracellular neurofibrillary tangles, which initiate oxidative stress, neuroinflammation, and lipid metabolism dysregulation, resulting in synaptic dysfunction, neurodegeneration, and neuron death in the brain (3,4). Clinically, AD patients experience episodic memory deficits in the early stage of disease and gradually progress to cognitive impairment and declined independent self-care abilities for years (5). Despite the advancements in the development of novel biomarkers, imaging tools, therapeutic drugs, and patient care in the past decade, the prognosis of AD patients remains dismal due to the lack of clarity about mechanisms underlying AD pathology (2). Therefore, the elucidation of the mechanism underlying AD development and progression might fuel the investigation of potential treatment targets for slowing the progression of AD or even curing AD.

MicroRNAs (miRNAs) consist of a class of endogenous short non-coding RNA molecules with approximately 19 to 25 nucleotides in length, which are proposed as essential regulators for gene expression at the post-transcriptional level via targeting mRNA for degradation or translation inhibition (6,7). miRNAs are reported to participate in the regulation of neuronal differentiation, dendrite spine morphology, and synaptic plasticity (8,9). Among these identified miRNAs, microRNA (miR)-146a is reported to be upregulated in brain regions targeted by AD neuropathology such as the superior temporal lobe neocortex and hippocampus (10). Furthermore, one study showed that miR-146a is upregulated in the AD brain and mediates the modulation of complement factor H (CFH) that is involved in the inflammatory response regulation (11). Another study showed that miR-146a induces cell apoptosis by suppressing the low density lipoprotein-related protein 2 (Lrp2)/Akt in SH-SY5Y cell lines (12). In addition, miR-146a/STAT1/MYC pathway is identified to be essential for regulating the progression of AD by bioinformatics analysis (13). On these bases, we conducted this study with the aim to explore the effect of miR-146a inhibition on regulating cell apoptosis, total neurite outgrowth, inflammation, and STAT1/MYC pathway in cellular AD models.

## Material and Methods

### Cell culture

Rat pheochromocytoma (PC12) cell line was obtained from Cell Resource Center of Shanghai Institute of Life Sciences, Chinese Academy of Sciences (China) and cultured in DMEM medium (Gibco, USA) containing 5% fetal bovine serum (FBS) (Gibco), 10% horse serum (Gibco), 100 IU/mL penicillin, and 100 μg/mL streptomycin (Gibco). The PC12 cells were maintained at 37°C in a 95% humidified incubator with 5% CO_2_. Rat primary cortical neurons were isolated from the brain cortex of Sprague Dawley rat embryos on embryonic day 16. The rat was purchased from Shanghai Lab Research Center (China), and the isolation and culture of rat embryo primary cortical neurons were performed according to the methods described in a previous study (14). The animal experiments were approved by the Institutional Animal Care and Use Committee of Huangshi Central Hospital and were conducted in line with the guidelines of the Care and Use of Laboratory Animals. Experiments were performed in biological triplicates, with only one technical replicate each.

### Cellular AD model construction

For the PC12 cellular AD model construction, oligomerized Aβ1-42 was prepared beforehand. Aβ1-42 (Sigma, USA) was dissolved in dimethyl sulfoxide (DMSO) (Sigma) to a final concentration of 1 mM, sealed in an EP tube, and was incubated at 37°C for 7 days to promote aggregation. Following incubation, the Aβ1-42 DMSO solution was diluted to 1 μM in serum-free medium for subsequent use. Induction differentiation by nerve growth factor (NGF) was then performed. After discarding the initial medium, the PC12 cells were incubated in DMEM medium (Gibco) supplemented with 20 ng/mL NGF (Sigma) and 10% FBS (Gibco) for 72 h at 37°C. Finally, NGF-stimulated PC12 cells were treated with 1 μM oligomerized Aβ1-42 peptides for 24 h to construct the cellular AD model.

As for the cortical neuron cellular AD model construction, the rat embryo primary cortical neurons were also treated with 1 μM oligomerized Aβ1-42 peptides for 24 h. After cellular AD model construction, using cells without Aβ1-42 treatment as control, the cell viability in PC12 and cortical neuron cellular AD models was assessed using the Cell Counting kit-8 (CCK-8, Dojindo, Japan) in accordance with manufacturer's manual. miR-146a expressions in both cellular AD models were determined by reverse transcription-quantitative polymerase chain reaction (RT-qPCR).

### Transfection

The miR-146a inhibitor and negative-control inhibitor were purchased from Guangzhou RiboBio Co., LTD. (China). The PC12 and cortical neuron cellular AD models were transfected with miR-146a inhibitor and negative-control (NC) inhibitor, respectively, by Lipofectamine 2000 (Invitrogen, USA); the cells were termed as miR-inhibitor group and NC-inhibitor group, respectively. miR-146a expressions in both groups were detected by RT-qPCR after 24-h incubation at 37°C.

### Cell apoptosis detection

At 48 h after transfection, cell apoptosis in both groups was detected by Hoechst/PI assay with the use of Hoechst 33342 (Sigma) and propidium iodide (PI) (Sigma). The procedures of Hoechst/PI assay were performed according to a previous study (15). Graphics were obtained using an inversion fluorescence microscope (Leica, Germany), and the cell apoptosis rate was calculated using the damaged cells divided by the total cells in the visual field.

### Total neurite outgrowth detection

Cellular morphology and neurite outgrowth were observed with a microscope (Leica, Germany) at 48 h after transfection. The neurite outgrowth was quantified using Imaging software Presage (Advanced Imaging Concepts, Inc., USA), and the total neurite outgrowth per cell was calculated using total length of neurite outgrowth of cells divided by total cell counts in the visual field (15).

### Inflammatory cytokines detection

Supernatant in both groups was collected at 48 h after transfection, and the inflammatory cytokines including tumor necrosis factor α (TNF-α), interleukin (IL)-1β, IL-6, and IL-17 in the supernatant were detected using commercial enzyme-linked immune-sorbent (ELISA) kits (Sigma-Aldrich, USA) following the manufacturer's manuals.

### Pathway detection

According to a previous study, the miR-146a/STAT1/MYC pathway may play a crucial role in AD (13). Consequently, to validate whether the STAT1/MYC was regulated by miR-146a in AD models, we detected the STAT1 and c-Myc expression in both groups using RT-qPCR and western blot assay at 48 h after transfection. Furthermore, to validate the hypothetical explanation (whether AKT/p-AKT was regulated by miR-146a in AD models), we detected the AKT and phosphorylated AKT (p-AKT) protein expressions in both groups using western blot assay. The western blot assay was carried out according to the procedures described in a previous study (15). In brief, RIPA Buffer (Sigma) was used for protein extraction. Pierce™ BCA Protein Assay kit (Thermo Scientific, USA) was used for protein quantification, anti-STAT1 antibody (1:1000 dilution, Abcam, UK), anti-c-Myc antibody (1:1000 dilution, Abcam), anti-AKT antibody (1:500 dilution, Abcam), and anti-p-AKT antibody (1:500 dilution, Abcam) were used as the primary antibody, and goat anti-rabbit IgG H&L (HRP) (1:10000 dilution, Abcam) was used as the secondary antibody. Novex™ ECL Chemiluminescent Substrate Reagent kit (Invitrogen) was used for chemiluminescence. RT-qPCR was performed as described below.

### RT-qPCR

Total RNA was obtained from the PC12 cells and rat primary cortical neurons by RNeasy Protect Mini kit (Qiagen, Germany), and then was reversely transcribed into complementary DNA (cDNA) by ReverTra Ace^®^ qPCR RT Master mix (Toyobo, Japan). Subsequently, PCR amplification of cDNA was conducted by SYBR^®^ Green Realtime PCR Master mix (Toyobo). The relative expressions of miR-146a, STAT1, and c-Myc were normalized by 2^-△△Ct^ method; U6 was used as an internal reference for miR-146a and GAPDH was used as an internal reference for both STAT1 and c-Myc. Of note, the same amount of RNA was used for the miR-146a detection in each group (control, Aβ1-42 treated, NC-inhibitor, and miR-inhibitor groups) to balance different cell number. The applied primer sequences were as follows: miR-146a (miR-146a-3p), F: 5′-ACACTCCAGCTGGGACCTGTGAAGTTCAGT-3′, R: 5′-TGTCGTGGAGTCGGCAATTC-3′; U6, F: 5′-GCTTCGGCAGCACATATACTA-3′; R: 5′-ATGGAACGCTTCACGAATTTGC-3′; STAT1: F: 5′-TTGTGGAGTGGAAGCGAAGAC-3′; R: 5′-TGCTGGAAGAGGACGAAGGT-3′; c-Myc: F: 5′-GCTCTGCTCTCCGTCCTATGT-3′, R: 5′-CAGTCCTGGATGATGATGTTCTTGA-3′; GAPDH: F: 5′-GAGTCCACTGGCGTCTTCAC-3′; R: 5′-ATCTTGAGGCTGTTGTCATACTTCT-3′.

### Statistical analysis

Data are reported as bar plots (means±SD) and analyzed by the GraphPad Prism 7.01 software (GraphPad Inc., USA). Comparison between two groups was determined by the unpaired *t*-test. P <0.05 indicated statistical significance.

## Results

### Comparison of cell viability and miR-146a relative expression after Aβ1-42 treatment

**Figure 1:**
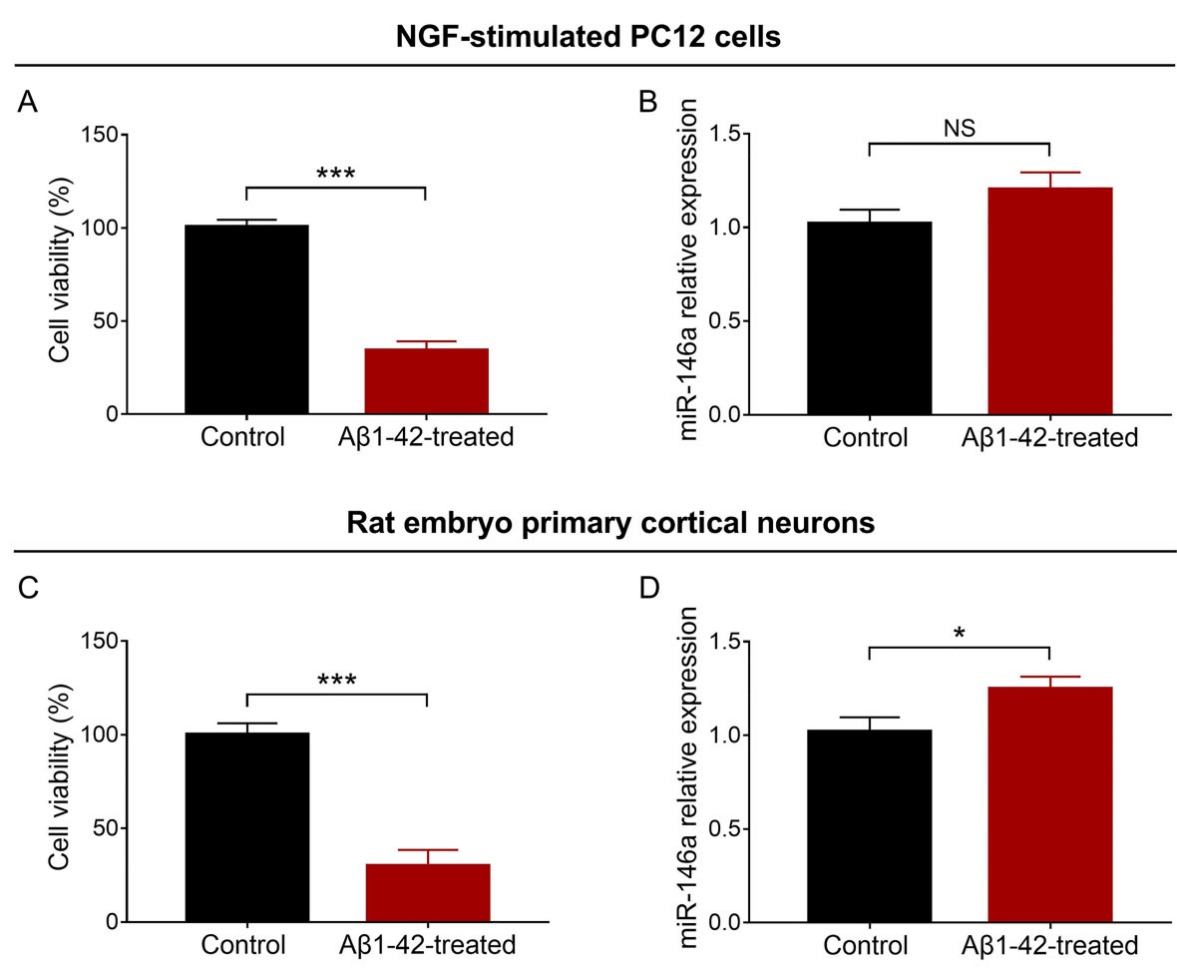
Comparison of cell viability (**A**) and miR-146a relative expression (**B**) between control and Aβ1-42-treated groups in the NGF-stimulated PC12 cellular AD model and in the rat embryo primary cortical neuron cellular AD model (**C** and **D**). miR: microRNA; AD: Alzheimer's disease; NGF: nerve growth factor; NS: not significant. Data are reported as means±SD. *P<0.05, ***P<0.001 (*t*-test).

In NGF-stimulated PC12 cells, cell viability was decreased in the Aβ1-42-treated group compared with the control group (P<0.001) (Figure 1A). No difference of miR-146a relative expression was observed between the Aβ1-42-treated group and control group (P>0.05) (Figure 1B). As for rat embryo primary cortical neurons, cell viability was attenuated in the Aβ1-42-treated group compared with the control group (P<0.001) (Figure 1C) and miR-146a relative expression was increased in the Aβ1-42-treated group compared with the control group (P<0.05) (Figure 1D).

### miR-146a relative expression and cell viability after transfection

**Figure 2:**
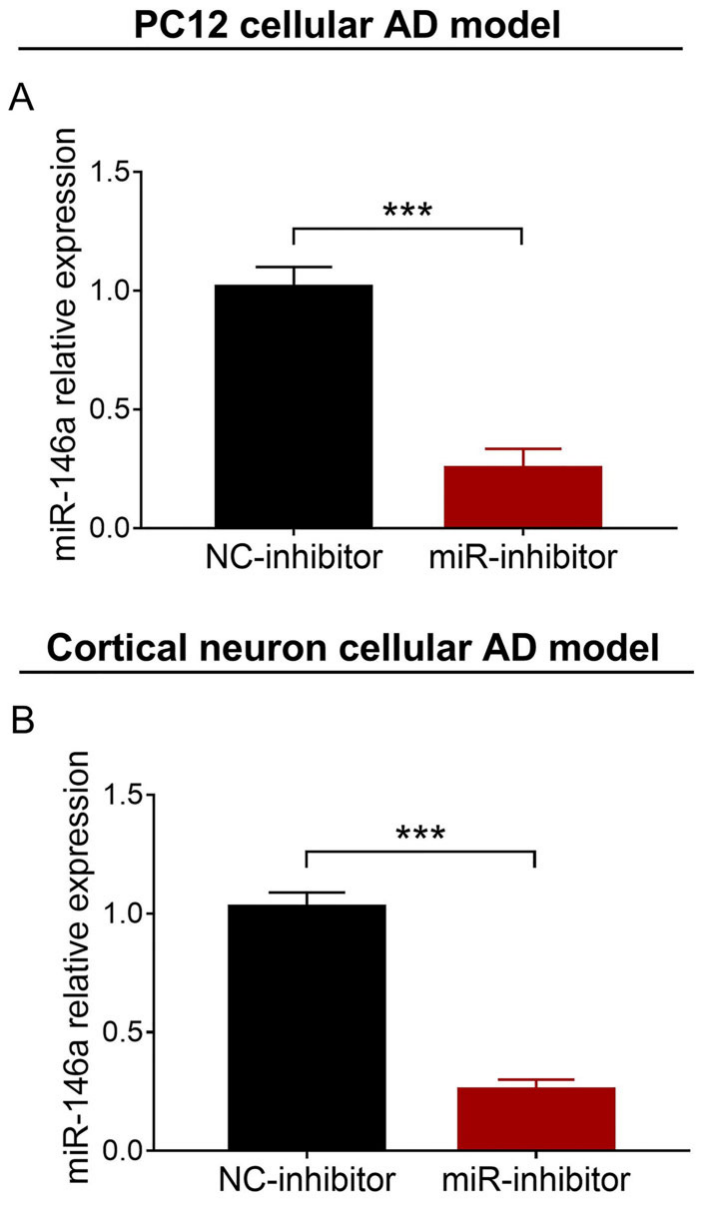
Comparison of miR-146a relative expression between NC-inhibitor group and miR-inhibitor group in PC12 (**A**) and cortical neuron (**B**) cellular AD models. miR: microRNA; NC: negative control; AD: Alzheimer's disease. Data are reported as means±SD. ***P<0.001 (*t*-test).

After transfection, miR-146a relative expression was greatly reduced in the miR-inhibitor group compared with the NC-inhibitor group in the PC12 cellular AD model (P<0.001) (Figure 2A). miR-146a relative expression was also significantly lower in the miR-inhibitor group than in the NC-inhibitor group in the cortical neuron cellular AD model (P<0.001) (Figure 2B). Cell viability was not different between the miR-inhibitor group and the NC-inhibitor group in both PC12 (P>0.05) (Supplementary Figure S1A) and cortical neuron cellular AD models (P>0.05) (Supplementary Figure S1B).

### miR-146a inhibition reduced cell apoptosis

**Figure 3:**
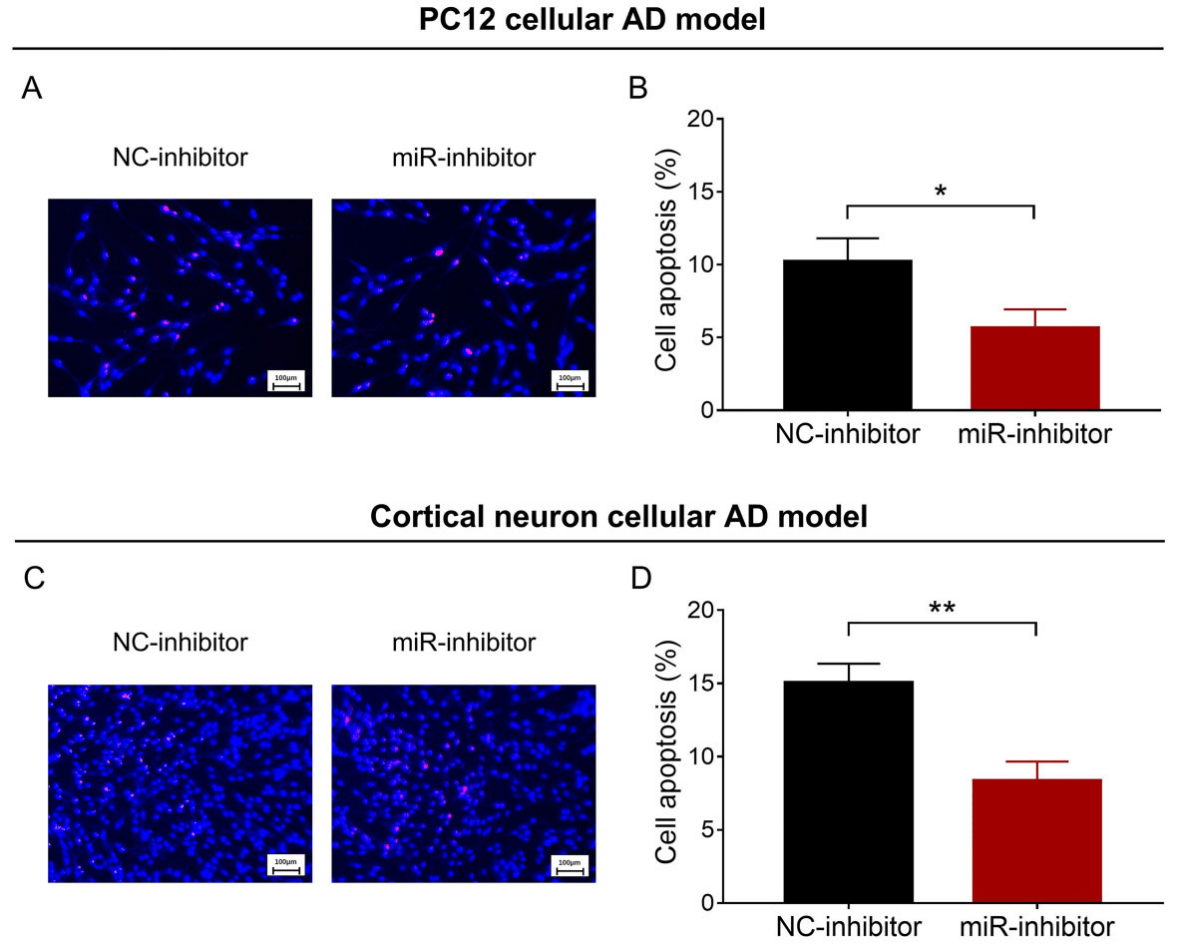
Cell apoptosis in NC-inhibitor group and miR-inhibitor group in PC12 (**A** and **B**) and cortical neuron (**C** and **D**) cellular AD models. Blue color indicates Hoechst-stained cells and red color indicates PI-stained cells (scale bar: 100 μm). miR: microRNA; NC: negative control; AD: Alzheimer's disease; PI: propidium iodide. Data are reported as means±SD. *P<0.05, **P<0.01 (*t*-test).

In PC12 and cortical neuron cellular AD models, cell apoptosis was attenuated in the miR-inhibitor group compared with the NC-inhibitor group (P<0.05) (Figure 3A-D).

### miR-146a inhibition promoted total neurite outgrowth

**Figure 4:**
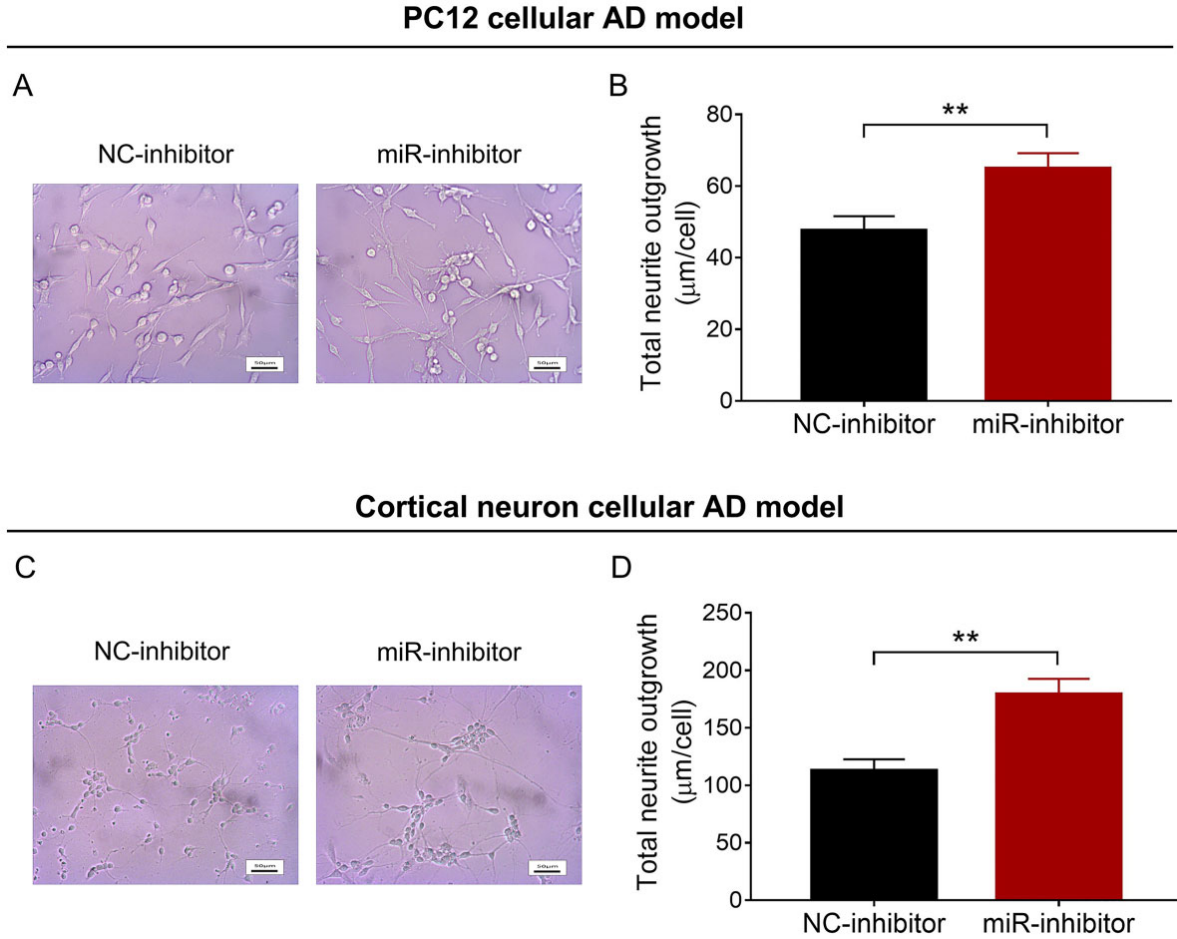
Total neurite outgrowth in NC-inhibitor group and miR-inhibitor group in PC12 (**A** and **B**) and cortical neuron (**C** and **D**) cellular AD models (scale bar: 50 μm). miR: microRNA; NC: negative control; AD: Alzheimer's disease. Data are reported as means±SD. **P<0.01 (*t*-test).

In PC12 and cortical neuron cellular AD models, total neurite outgrowth was increased in the miR-inhibitor group compared with the NC-inhibitor group (P<0.01) (Figure 4A-D).

### miR-146a inhibition decreased inflammation

**Figure 5:**
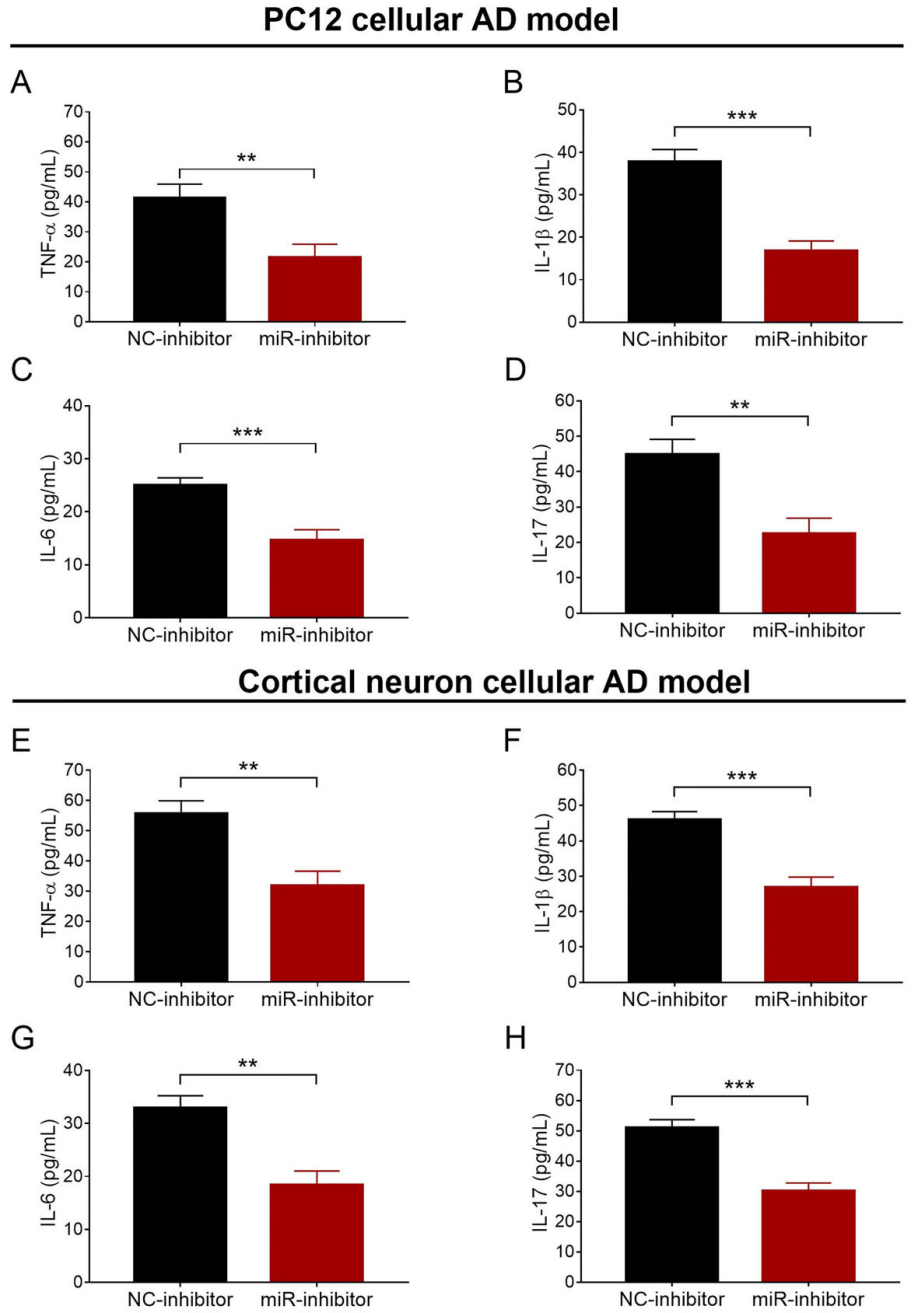
Comparison of supernatant TNF-α, IL-1β, IL-6, and IL-17 between NC-inhibitor group and miR-inhibitor group in PC12 (**A**-**D**) and cortical neuron (**E**-**H**) cellular AD models. miR: microRNA; TNF: tumor necrosis factor; IL: interleukin; NC: negative control; AD: Alzheimer's disease. Data are reported as means±SD. **P<0.01, ***P<0.001 (*t*-test).

In both PC12 and cortical neuron cellular AD models, supernatant TNF-α (P<0.01) (Figure 5A and E), IL-1β (P<0.001) (Figure 5B and F), IL-6 (P<0.001) (Figure 5C and G), and IL-17 (P<0.01) (Figure 5D and H) levels were attenuated in the miR-inhibitor group compared with the NC-inhibitor.

### miR-146a inhibition suppressed STAT1/MYC pathway

**Figure 6:**
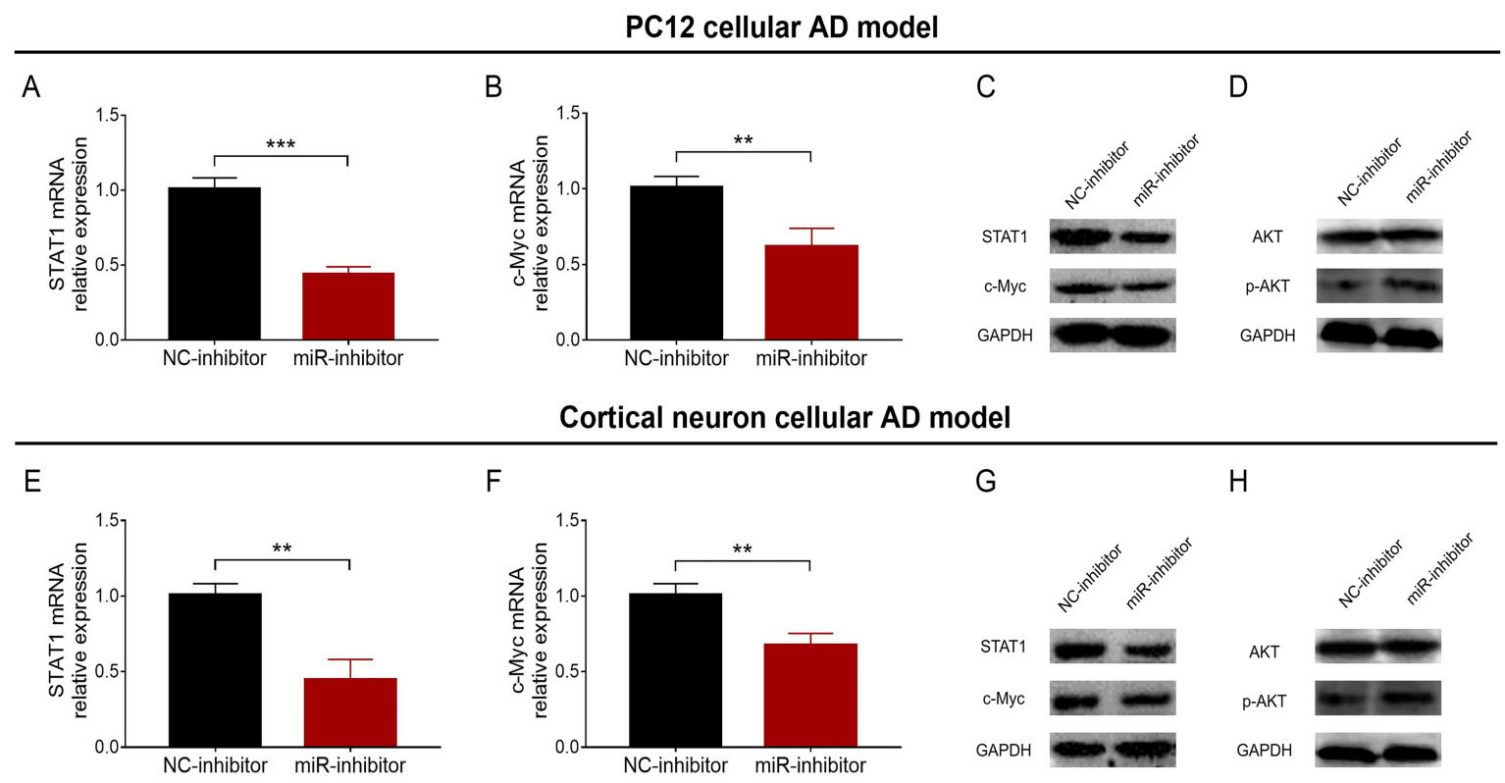
Comparison of STAT1 mRNA (**A**) and c-Myc mRNA (**B**) relative expressions and STAT1 and c-Myc protein expressions (**C** and **D**) between NC-inhibitor group and miR-inhibitor group in PC12 cellular AD model. Comparison of STAT1 mRNA (**E**) and c-Myc mRNA (**F**) relative expressions and STAT1 and c-Myc protein expressions (**G** and **H**) between NC-inhibitor group and miR-inhibitor group in cortical neuron cellular AD model. miR: microRNA; STAT1: signal transducer and activator of transcription 1; NC: negative control; mRNA: messenger RNA; AD: Alzheimer's disease; p-AKT: phosphorylated-AKT. Data are reported as means±SD. **P<0.01, ***P<0.001 (*t*-test).

In the PC12 cellular AD model, STAT1 (P<0.001) and c-Myc (P<0.01) mRNA and protein expressions were reduced in the miR-inhibitor group compared with the NC-inhibitor group (Figure 6A, B, and C). In the cortical neuron cellular AD model, STAT1 (P<0.01) and c-Myc (P<0.01) mRNA and protein expressions were also lower in the miR-inhibitor group than in the NC-inhibitor group (Figure 6E, F, and G). In the PC12 cellular AD model, p-AKT protein expression was increased, while AKT protein expression did not differ in the miR-inhibitor group compared with the NC-inhibitor group (Figure 6D). In the cortical neuron cellular AD model, the findings were similar (Figure 6H).

## Discussion

From the present study, we determined that miR-146a inhibition promoted total neurite outgrowth and suppressed cell apoptosis and inflammation as well as the STAT1/MYC pathway in both PC12 and cortical neuron cellular AD models.

AD, a complex age-related neurodegenerative disorder, is marked by two core pathological protein abnormalities, toxic Aβ aggregation and hyperphosphorylated tau protein, inducing loss of connections between cells and extensive neurodegeneration over time (16). In addition to the well-studied Aβ and tau pathology, lifestyle, vascular risk factors, and genetic susceptibility have been proposed as major contributors to the complex etiology of AD (3). However, the genetic mechanisms underlying AD are still poorly understood. Following the development and progress in the genome-wide association studies and next-generation sequencing in recent years, extensive research on microRNA has provided new insights into the pathogenesis of neurodegenerative diseases (9). It has been reported that many miRNAs express abundantly in the central nervous system and play a vital role in regulating neuron differentiation, synaptic plasticity, neurite outgrowth, and neurodegeneration (8,9).

As one of the most extensively studied miRNAs, miR-146a is proposed as a key regulator of the innate immune and inflammatory responses in certain immunological and brain cell types, which has been implicated in the pathogenesis of AD (12,17,18). For instance, miR-146a triggers the downregulation of interleukin-1 receptor-associated kinase (IRAK)-1 and compensatory upregulation of IRAK-2, which drives intensified inflammatory responses in the hippocampus and neocortex of the AD brain and stress human astroglial cells in primary culture (18). Another study, based on human neuroblastoma SH-SY5Y cells, reveals that miR-146a inhibits Lrp2 protein expression and reduces subsequent Akt activation as well as pro-apoptotic caspases-3 induction, which ultimately elevates cell apoptosis in AD (12). In addition, miR-146a is associated with senile plaque density and synaptic pathology in Tg2576 and in 5xFAD transgenic mouse models (17).

Our results were in line with this aforementioned evidence, which showed that cell apoptosis and inflammation (reflected by supernatant TNF-α, IL-1β, IL-6, and IL-17 levels) were decreased in miR-146a-inhibitor-treated cells compared with NC inhibitor-treated cells in both PC12 and cortical neuron cellular AD models. Herein, we proposed several explanations. First, miR-146a inhibition might have enhanced the translation of downstream proteins (e.g., low-density lipoprotein receptor-related protein-2), which mediated the activation of AKT, the induction of pro-apoptotic caspase-3, and suppressed cell apoptosis in both AD models (12). Meanwhile, miR-146a inhibition increased p-AKT protein expression, and it did not change AKT protein expression in either cellular AD model, which supported our hypothetical explanation that miR-146a inhibition might induce the activation of AKT in cellular AD models. Second, miR-146a inhibition might have facilitated specific inflammation-relevant mRNAs such as CFH, interleukin-1 receptor-associated kinase 1, and tetraspanin-12, which inhibited the release of pro-inflammatory cytokines and decreased sustained inflammation in both AD models extensively (11,18).

Furthermore, we also observed that total neurite outgrowth was elevated in the miR-146a inhibitor-treated cells compared with the NC inhibitor-treated cells in both cellular AD models. The possible reason could be that miR-146a inhibition might have upregulated the expression of certain brain-essential mRNA targets (e.g., amyloid precursor protein), which increased the neurite outgrowth in the cellular AD models (19,20). However, this speculation needs further validation.

In a previous study, consistent differentially expressed genes (cDEGs) were obtained by a meta-analysis of multiple gene microarray datasets, and differentially expressed miRNA (DEmiRs) were retrieved from one miRNA expression profile (GSE16759). Then, the potential active transcription factor (TF)-miRNA regulatory subnetwork in AD was obtained by mapping the cDEGs and DEmiRs to the curated TF-miRNA regulatory network (constructed by integrating TRANSFAC, TransmiR, miRTarBase, miRecords and TarBase data resources) as active seed nodes and connecting active seed nodes with their immediate neighbors. Next, potential active TF-miRNA pathways in AD were found by breadth-first-search method. Lastly, according to the known AD-related genes and miRNAs (derived from the GeneCards database), nine active TF-miRNA pathways in AD were identified by hypergeometric test, among which miR-146a/STAT1/MYC is the source of all nine pathways and may play a critical role in the progression of AD (13). Thus, the present study evaluated the effect of miR-146a inhibition on regulating STAT1/MYC pathway in AD. We found that STAT1 and c-Myc mRNA and protein expressions were decreased in the miR-146a inhibitor-treated cells compared with NC inhibitor treated cells in both cellular AD models, which implied that the miR-146a inhibition suppressed the STAT1/MYC pathway in AD. In addition, it might also explain the effect of miR-146a inhibition on suppressing cell apoptosis and inflammation and enhancing total neurite outgrowth in both AD models as follows: STAT1 is known to induce inflammation by increasing the expression of pro-inflammatory mediators and to mediate cell apoptosis by triggering the transcriptional activation of death-modulating genes (e.g., caspases, death receptors, and ligands) (21,22). Meanwhile, MYC might induce neurodegeneration via activating the cell cycle in the neurons (23). Additionally, suppression of STAT1 was reported to be accompanied by decreased MYC expression (22). Combining the aforementioned evidence and the findings of our cellular experiments, miR-146a inhibition might attenuate cell apoptosis and inflammation and elevate total neurite outgrowth through suppressing the STAT1/MYC pathway. However, our speculation requires further verification. Due to the limited budget, only cellular AD models were used in the investigations, thus, further animal study is needed to validate our results.

In conclusion, miR-146a inhibition promoted total neurite outgrowth and suppressed cell apoptosis, inflammation, as well as STAT1/MYC pathway in PC12 and cortical neuron cellular AD models, which could indicate miR-146a as a potential therapeutic target for AD.

## Supplementary Material

Click here to view [pdf].
